# Electricity and Life

**Published:** 1875-04

**Authors:** 


### ELECTRICITY AND LIFE.

Electricity—about which so little is really known, the wonderful possibilities of which have scarcely begun to be developed—has been lifted, by scientific investigation, high above the region of charlatanism, and placed upon the list of positive remedial agents. Why the electrical current, when passed from the nerve-centres along the various branches, is competent to reduce irritation and allay pain, we can only conjecture. The fact exists, however, and the sufferer is more nearly interested in the fact than in the cause. No phenomena are more closely investigated than the electrical, and none are the subjects of wider discussion. The question "Is Electricity Life?" has been answered affirmatively by many of the leading scientific minds of Europe; and the researches of the most eminent electricians substantiate the fact that a current, precisely akin to that generated by the decomposition of metals, exists in all our muscles and nerves; and, further, that in disease, this electrical force is essentially diminished.

The unavoidable conclusion from this state of things, is, therefore, that electricity is not only life, but life at its best; life, vigorous and unimpaired; and that this energetic, vigorous life, can only be maintained by supplying whatever of electrical force has been displaced or exhausted by incorrect habits of life, by exposure, or exhaustion.

The one problem to be solved is this: Shall this subtle fluid, so potent for good if wisely employed, be applied through the poles of the battery, as an electrical "shock," so-called, or shall we seek the benefit desired, through the continuous current, by wearing upon the portion to be revitalized, such a combination of metals as shall insure a constant though mild electrical action?

The truth is, each method has its specific place—a fact well understood by many medical men. That the simple form—the primary current, induced not by the positive decomposition of metals, but rather by moist contact—possesses certain advantages, we are convinced; that it is safe in unskillful hands is a fact established. The advantages rest in the fact that the current never ceases so long as the appliance is worn; and therefore that the result is cumulative rather than interrupted and spasmodic, and that no unpleasant sensations attend its use.

The best results, so far as we can learn, have attended the use of the electrical appliance invented by Mr. E. J. Seibert, No. 28 Barclay Street, New York, and which he has named "The Voltaic Armadillo." It is a combination of metals, constructed on the plan of the voltaic pile. The alternating sheets of appropriate metals are finely attached to morocco, overlapping, as do the scales of a fish, and securing perfect flexibility. The plates are light, and no inconvenience is experienced by the wearer. The current is very perceptible, and is greatly increased on an accession of the perspiratory secretion, particularly if the perspiration is rendered acrid by diseased action.

This "Armadillo" is a very simple affair; and in this simplicity great merit lies. It is constructed in the form of bands of varying size, for the wrist, the arm, the thigh, the waist, the knee, the head, and the ankles; while inner soles, made in like manner, are provided for the feet. The price is very moderate, varying from one to five dollars.

Our chief object in thus alluding to this matter, is, to assure our readers that the use of these appliances in diseased conditions is based upon scientific truth, and that the highest medical authority places their employment far above and beyond the realm of charlatanry. We have tried them with good results. That they are competent to solace many of the ills of life can not be denied; and that entire safety attends their use, is everywhere admitted. A multitude of prominent physicians prescribe them, and discover benefits from their use.

If our readers would learn more concerning these curative agents, they can address the manufacturer, whose name we have given above, and he will forward much interesting literature concerning them.

